# Antifungal Activity of Neem Leaf Extract With Eucalyptus citriodora Oil and Cymbopogon martini Oil Against Tinea Capitis: An In-Vitro Evaluation

**DOI:** 10.7759/cureus.59671

**Published:** 2024-05-05

**Authors:** Rupali H Tiple, Shamli R Jamane, Deepak S Khobragade

**Affiliations:** 1 Pharmacy, Datta Meghe College of Pharmacy, Datta Meghe Institute of Higher Education and Research, Wardha, IND; 2 Bio-processing and Herbal Division, Mahatma Gandhi Institute for Rural Industrialization, Wardha, IND

**Keywords:** neem leaves extract, terpenoids, minimum inhibitory concentration, tinea capitis, antifungal spectrum

## Abstract

Introduction

Tinea capitis, often known as ringworm of the scalp, is a fungal infection that affects the scalp, eyelashes, and eyebrows. It is generally caused by dermatophytes from the genera *Trichophyton* and *Microsporum. Trichophyton tonsurans *and *Microsporum canis* are the main etiological agents responsible for most of the cases of tinea capitis globally. Tinea capitis commonly manifests as itchy, scaly patches of hair loss. Tinea capitis is the prevailing dermatophyte illness among children globally.

Methods

An in-vitroevaluation study was conducted to assess the antifungal properties of ethanolic extracts of neem leaves and the oils of *Eucalyptus citriodora* and *Cymbopogon martini*, both individually and in combination. The agar-well diffusion method and the M38-A2 microbroth dilution method were employed to evaluate the antifungal efficacy against pathogenic dermatophyte strains, namely *Microsporum canis* and *Trichophyton tonsurans*. The fully mature green leaves were treated with ethanol to make the neem leaf extract. Additionally, high-performance liquid chromatographic analysis was carried out to determine the contents of the terpenoids. Fluconazole, an antifungal drug, is used as a standard.

Results

The findings demonstrated an overall inhibition of the growth of dermatophytes at a minimal inhibitory concentration of 187.5 and 375 μg/ml for neem leaf extract and 0.625 to 2.5 μl/ml for selected herbal oils, whereas it was 0.25 μg/ml and 0.50 μg/ml for positive control against *Microsporum canis *and *Trichophyton tonsurans, *respectively.

Conclusion

The phytochemical investigation of the ethanolic extracts in neem leaves revealed the presence of terpenoids, which are known for their significant biological activity. The study's findings demonstrated the therapeutic capabilities of neem leaf extract in combination with the oils of *Eucalyptus citriodora* and *Cymbopogon martini *for managing the tinea capitis infection. A broader and improved antifungal spectrum was seen when neem leaf extract and oils were combined. Therefore, it can be developed into a suitable formulation for the management of tinea capitis.

## Introduction

Dermatophytosis is a prevalent cause of dermatology consultations on a global scale. Fungal infections affecting nails and skin are prevalent in 20-25% of the global population, with a particularly high occurrence of up to 40% in tropical and subtropical countries. This is mostly due to the prevailing warmth and humidity conditions. Tirado-Sánchez et al. [1] and Weitzman et al. [2] described dermatophytes based on their host preferences. Dermatophytes can be either anthropophilic (found on humans), zoophilic (found on animals), or geophilic (found in soil). In humans, the scalp is a common site of fungal infection. The anthropophilic fungi, like *Trichophyton schoenleinii*,* Trichophyton soundanense*, *Trichophyton tonsurans*,* Trichophyton rubrum*, *Epidemophyton floccosum*, and *Trichophyton violaceum* are some of the examples. Infected animals, especially stray cats and dogs, and small pets like rabbits, puppies, and kittens are the most common vectors for animal-parasitic fungal infections of the scalp. Zoophilic fungi such as *Microsporum distortum*, *Trichophyton mentagrophytes *var. interdigitale, *Trichophyton equinum*, *Microsporum canis*, *Microsporum nanum*, and *Microsporum ferrugineum* are examples. *Microsporum gypseum* is an example of a geophilic fungus that infrequently causes tinea capitis. The fungus species responsible for this phenomenon differs based on the geographical region and may undergo changes throughout time. Tinea capitis can be caused by a direct contact with the infected person or sharing utensils with a diseased person. It usually causes itchy, scaly, bald patches on the head. If left untreated, it may progress to form kerion, black dot, and favus. Kaur et al. [3] described the structure of hair. It has a cuticle, cortex, and medulla that make up its shaft. The outermost layer, called the cuticle, shields the innermost structure of the hair. The cortex is the mid-layer of the hair shaft that determines the hair's strength, texture, and colour. The medulla, or "marrow" of the hair, is the deepest layer. Fungi penetrate through the hair shaft of the outer root sheath of the hair follicle. When fungus infiltrates the shaft of the hair, it inhibits normal functions, leading to a tinea capitis infection. This infection can be non-inflammatory (without permanent hair loss) or inflammatory (causing painful nodules with pus and permanent hair loss). Decreased sebum production leads to a reduction in fatty acids and an increase in the scalp's pH, promoting the colonisation of dermatophytes and subsequent fungal illness. Tinea capitis is primarily observed in school-going children. Some individuals who have tinea capitis caused by *Trichophyton tonsurans* may carry a dormant infection in their scalp and shed viable fungal spores for a long time. In symptomatic adults, *Trichophyton tonsurans* is often associated with tinea corporis, while occurrences of tinea manuum and onychomycosis are rare. Tinea corporis and tinea capitis, caused by *Trichophyton tonsurans* and other pathogens, may be more prevalent in individuals, especially mothers, who have a close contact with children as compared to other adults. *Trichophyton tonsurans* is currently the predominant cause of tinea capitis in most of the cases in the United States, the United Kingdom, Brazil, Jamaica, and certain regions of Western Europe. On the other hand, *Microsporum canis* is the primary cause of tinea capitis in South America, Southern and Central Europe, Africa, the Middle East, and Western Asia. According to the findings of Leung et al. [4] and Gupta et al. [5], the main pathogens predominantly involved are anthropophilic and zoophilic species of the *Trichophyton *and *Microsporum *genera. According to Nagabhushan et al. [6], prevalent fungal infections include tinea corporis, tinea pedis, tinea cruris, tinea capitis, and tinea unguium. The incidence of dermatomycoses has increased due to a notable increase in immunocompromised status of the population in recent times. Allopathic antifungal medications have restricted effectiveness and may lead to side effects, such as hepatotoxicity, GI disturbances, cutaneous rashes, and leukopenia, in certain people undergoing treatment. The efficacy of contemporary antifungal drugs, such as azole derivatives, morpholines, and allyl amines, is commonly employed for the management of dermatomycoses and is compromised due to the emergence of fungal resistance, prolonged treatment duration, increased cost, and recurrence of infection if left untreated properly [3]. The phytochemistry of innumerable plant species has evidenced that phytochemicals may be a better source of antifungal medicine as compared to synthetically produced drugs. The process of extracting naturally occurring compounds with antifungal properties is becoming increasingly popular. Among these, terpenoids have demonstrated fungistatic or fungicidal properties against certain harmful fungi [3]. The neem tree, scientifically known as* Azadirachta indica* of the family *Meliaceae*, has gained global recognition due to its extensive array of medical attributes. According to Govindachari et al. [7], neem leaf components have shown anti-inflammatory, antihyperglycemic, antiulcer, antimalarial, antifungal, antibacterial, immunomodulatory, antioxidant, antimutagenic, and anticarcinogenic effects. The extensive research by Salazar et al. [8] on neem has been focused on its recognised antifungal potential. While Prasad et al. [9] analysed the combined effects of *Cymbopogon ambrosioides* and *Cymbopogon martini* oils on dermatophytes in male Guinea pigs by in-vivo evaluation. They found that oils from these two species, when used together, could potentially be used as a natural substitute for synthetic antifungal medications for treating tinea corporis (ringworm) and other superficial fungal infections in humans. According to the findings of Leticia et al. [10] and Tolba et al. [11], *Eucalyptus citriodora* oil exhibited greater antifungal action compared to *Eucalyptus globulus* and conventional medications against zoophilic fungi like *Candida albicans,* *Trichophyton mentagrophytes*, and *Microsporum gypseum*, while Luqman et al. [12] stated that the oil of *Eucalyptus citriodora *was* *found to be highly effective on *Trichophyton rubrum*, drug-resistant mutants of *Candida albicans*, and pathogenic bacteria.

The neem plant is considered a panacea in dentistry, particularly due to its extensive antimicrobial potency. Because of its extensive usage in hair and skin care products, it also has a significant impact on the cosmetics industry. As per the review of Alzohairy [13], the neem plant has a diverse range of active phytochemical compounds, such as triterpenoids (such as azadirachtin and nimbin), diterpenoids (such as nimbidine), glycosides, dihydrochalcone, tannins, coumarin, proteins, quercetin, carbohydrates, sulphurous chemicals, and polyphenolics. *Eucalyptus citriodora *contains citronellal, citronellol, and DL-isopulegol as its main constituents, along with other important compounds including limonene, P-cymene, alpha-pinene, geraniol, and camphene. *Eucalyptus citriodora* oil is commonly utilised for treating body aches, reducing fever, treating headaches, treating persistent bowel issues, and combating dysentery [11]. The essential oil derived from *Cymbopogon martini* consists mainly of trans-geraniol, geranyl acetate, b-elemene, linalool, and E-citral [9]. The other compounds include nerolidol, α-terpinene, terpinen-4-ol, and α-bisabolol. According to the findings of several earlier investigations, the antimicrobial efficacy of certain essential oils has been observed to improve when they are combined with certain other essential oils. Hence the combination of oils of *Eucalyptus citriodora*, *Cymbopogon martini, *and neem leaf extract was investigated further as a treatment for dermatophytosis.

## Materials and methods

The mature leaves of the neem plant were collected from the locality of Hindi Vishwa Vidyalaya, Wardha, Maharashtra, India. Specimens of herbarium were authenticated and deposited at the Department of Botany, Bajaj Science College, Wardha, Maharashtra. The leaves were thoroughly cleaned, dried in a shaded area for a duration of 14 days, and subsequently ground into a fine powder. Then 25 grams of neem leaf powder was treated with 100 ml of ethanol and soaked overnight. Following filtration, the suspension underwent centrifugation at 5000 rpm for 20 minutes. The liquid portion was evaporated in an aseptic area in glass Petri dishes under UV light. The high-performance liquid chromatography and phytochemical evaluation of neem leaf extract were carried out, and the dried neem leaf extract was stored at a temperature of -4°C. *Eucalyptus citriodora *oil and *Cymbopogon martini *oil were procured locally. The determination of antifungal activity was performed on two strains of dermatophytes, namely *Microsporum canis* (MTCC 2820) and *Trichophyton tonsurans* (MTCC 8475), procured from the Microbial Type Culture Collection and Gene Bank (MTCC), Chandigarh, India. The study used Saboraud's dextrose agar, modified Saboraud's dextrose agar, Saboraud's dextrose broth, potato dextrose agar, and dimethyl sulphoxide as control, and fluconazole as the standard. Freeze-dried fungi were activated in appropriate growth conditions on suitable media, as suggested by MTCC, Chandigarh, India.

All the measurements were replicated three times for each treatment, and the data were recorded in an Excel spreadsheet and presented as the mean ± standard deviation (SD). The statistical examination involved conducting a one-way analysis of variance (ANOVA) followed by Tukey's post hoc test. Significance was attributed to P values lower than 0.05 (p<0.05).

After being activated, the fungi were subcultured and incubated at about 27°C for seven to 12 days. The fungal inoculums were prepared by using freshly cultured fungal broth and adjusted to a turbidity of 0.5 McFarland units. The standardisation process was conducted using the UV visible spectrophotometer (Agilent Cary 100; Agilent Cary, Santa Clara, USA).

Determination of antifungal activity

Neem leaf extract was diluted with dimethyl sulphoxide (DMSO) at a 1 g/ml concentration. The neem leaf extract was further diluted to reach the required concentration of 50-500 μg/ml. The antifungal activity was evaluated using Saboraud's dextrose agar media by the agar-well diffusion method. The fungal inoculum was homogeneously distributed on agar plates using a sterilised glass spreader. Four wells, each with a diameter of 6 mm, were created on an agar plate using a sterile cork borer. The four wells were incorporated with 50 μl of neem leaf extract, with 50 to 500 μg/ml concentrations. The agar plates were incubated for seven days at 37°C. Furthermore, the antifungal activity of *Eucalyptus citriodora* and *Cymbopogon martini* oils was investigated on the dermatophytes under study. The inhibitory zone's diameter was measured by a Hi-Antibiotic Zone Reader (Prolab, Mumbai, India). Fluconazole and DMSO were used as a standard and a control, respectively. The experiment was repeated three times, and the results were recorded as the average of those three separate trials. Four combinations of neem leaf extract and selected oils were prepared based on the prior data interpretation, and a bioassay was conducted in triplicate.

Determination of minimum inhibitory concentration

The minimum inhibitory concentration (MIC) of neem leaf extract and selected oils was determined using 96-well microtitre plate by the microbroth dilution method. Dilutions were made in the range of 3000 to 2.92 µg/ml and from 80 to 0.078 µl/ml, respectively, for neem leaf extract and oils, respectively, in Sabouraud's dextrose broth previously standardised with fungal inoculum. The Sabouraud's dextrose broth served as the control, and fluconazole was used as a standard. The Sabouraud's dextrose broth, along with neem leaf extract and oils with stated dilutions, were added to a 96-well microtiter plate. The plate was then placed in a biological oxygen demand (BOD) incubator (REMI Cl 16, Mumbai, India) and incubated at a temperature of 37°C. Turbidity was observed for a period of seven days. The minimum inhibitory concentration was reported as the lowest concentration of extract or oil that did not exhibit any noticeable growth after seven days of incubation.

## Results

The antifungal bioassays demonstrated varying degrees of growth inhibition among the tested dermatophytes when exposed individually and in combination with neem leaf extract and selected oils. Tables 1, 2 denote significant differences in fungal growth inhibition based on the concentration of neem leaf extract and selected oils of *Eucalyptus citriodora* and *Cymbopogon martini.*

**Table 1:** Zone of inhibition for neem leaf extract and selected oils against Microsporum canis NLE=neem leaf extract; ECO=*Eucalyptus citriodora* oil; CMO=*Cymbopogon martini *oil; ZOI= zone of inhibition; STD=fluconazole (standard); CTRL=dimethyl sulphoxide (control). Data (mean ± SD) (n = 3). Different superscripts denote significant difference (P<0.05) and # shows insignificance (P > 0.05)

S. No.	Name of plant part/drug	Concentration	Zone of inhibition (mm)			Mean ZOI ± SD	F-value	P-value
1	NLE (µg/ml)	50	0.0	0.0	0.0	0.0± 0.0	6.86	0.06 (˃0.05)
		100	0.0	0.0	0.0	0.0± 0.0		
		150	7.0	7.4	7.2	7.2 ± 0.2^a#^		
		200	7.5	7.5	7.8	7.6 ± 0.17^a#^		
2	ECO (µl)	0.5	0.0	0.0	0.0	0.0± 0.0	23.58	<0.05
		0.6	7.2	7.5	7.5	7.4 ± 0.17^b^		
		0.7	7.5	7.8	7.7	7.67 ± 0.15^b^		
		0.8	8.1	8.3	8.2	8.2 ± 0.1^b^		
3	CMO (µl)	0.5	0.0	0.0	0.0	0.0± 0.0	111.80	<0.05
		1.0	13.2	13.8	13.5	13.5 ± 0.3^c^		
		1.5	15.5	16.2	16.5	16.07± 0.5^c^		
		2.0	18.2	18.5	19.0	18.57 ± 0.4^c^		
4	STD (µg/ml)	25	13.8	14.1	14.0	13.97±0.15	N/A	N/A
5	CTRL (µl)	50	0.0	0.0	0.0	0. 0± 0.0	N/A	N/A

**Table 2:** Zone of inhibition for neem leaf extract with selected oils against Trichophyton tonsurans NLE=Neem leaf extract; ECO=*Eucalyptus citriodora* oil; CMO=*Cymbopogon martini* oil; ZOI= Zone of Inhibition; STD=Fluconazole (standard); CTRL=Dimethyl sulphoxide (control). Data (mean ± SD) (n = 3): different superscripts denote significant difference (P<0.05)

S. No.	Name of plant part/drug	Concentration	Zone of inhibition (mm)			Mean ZOI ±SD	F-value	P-value
1	NLE (µg/ml)	350	0.0	0.0	0.0	0.0± 0.0	53.07	<0.05
		400	7.8	7.5	7.8	7.7 ± 0.17^a^		
		450	8.0	8.2	8.5	8.23±0.25^a^		
		500	9.5	10.0	10.2	9.9 ± 0.36^a^		
2	ECO (µl)	1.0	7.8	7.5	8.0	7.77 ± 0.25^b^	92.02	<0.05
		1.5	8.4	8.2	8.4	8.33 ± 0.12^b^		
		2.0	9.6	9.2	9.5	9.43 ± 0.21^b^		
		2.5	11.2	10.5	10.9	10.87±0.35^b^		
3	CMO (µl)	2.5	7.5	7.8	7.5	7.6 ± 0.17^c^	64.62	<0.05
		3.0	8.5	8.2	8.9	8.53 ± 0.35^c^		
		3.5	9.8	10.0	10.5	10.1 ± 0.36^c^		
		4.0	11.5	12.0	11.8	11.7± 0.25^c^		
4	STD (µg/ml)	50	9.5	10.1	9.8	9.8 ± 0.3	N/A	N/A
5	CTRL (µl)	50	0.0	0.0	0.0	0. 0± 0.0	N/A	N/A

**Figure 1:**
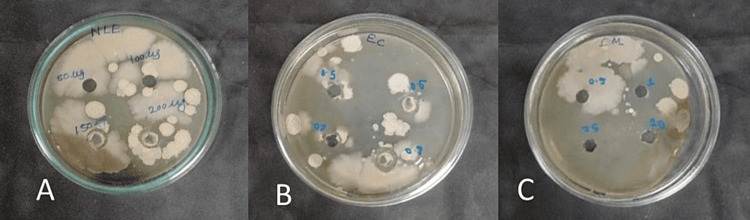
Antifungal effect of (A) neem leaf extract, (B) Eucalyptus citriodora oil, and (C) Cymbopogon martini oil on Microsporum canis

**Figure 2:**
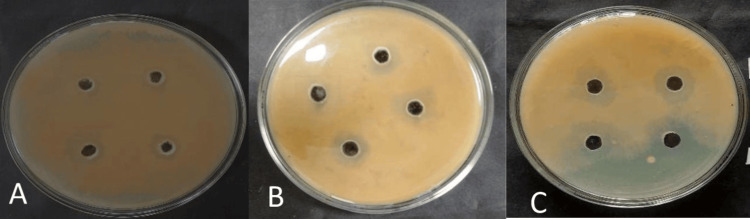
Antifungal effect of (A) neem leaf extract, (B) Eucalyptus citriodora oil, and (C) Cymbopogon martini oil on Trichophyton tonsurans

Figures 1, 2 show the clear zone of inhibition for individual effects of neem leaf extract and selected oils against *Microsporum canis* and *Trichophyton tonsurans*, respectively. 

 

 

Table 3 denotes the fungal inhibition of four different combinations of neem leaf extract and selected oils of *Eucalyptus citriodora *and *Cymbopogon *martini on *Microsporum canis* and* Trichophyton tonsurans,* respectively.

**Table 3:** Zone of inhibition for different combinations of neem leaf extract with selected oils against Microsporum canis NLE=Neem leaf extract, ECO=*Eucalyptus citriodora* oil, CMO=*Cymbopogon martini* oil, ZOI= zone of inhibition, STD=Fluconazole (standard), CTRL=dimethyl sulphoxide (control), * inhibition of the growth across the entire 90 mm plate. N/A=not applicable, data (mean ± SD) (n=4). Different superscripts denote a significant difference (P<0.05).

S. No.	Combination	NLE	ECO	CMO	ZOI (mm)			Mean ZOI±SD	F-value	P-value
1	Combination 1	50	0.5	0.5	10.5	10.8	10.2	10.5± 0.3^d^	350.22	<0.05
2	Combination 2	50	0.6	0.5	12.8	12	11.5	12.1 ± 0.66^e^		
3	Combination 3	50	0.7	1.0	20.2	19.5	20	19.9 ± 0.36^f^		
4	Combination 4	50	0.8	1.5	90	90	90	90± 0.00*		
5	STD (µg/ml)	25	N/A	N/A	13.8	14.1	14.0	13.97±0.15	N/A	N/A
6	CTRL (µl)	50	N/A	N/A	0.0	0.0	0.0	0.0 ± 0.0	N/A	N/A

**Figure 3:**
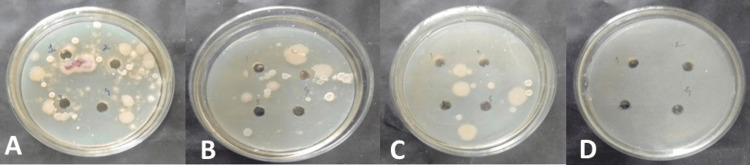
Antifungal activity of different combinations of neem leaf extract with Eucalyptus citriodora oil and Cymbopogon martini oil on Microsporum canis. A: Combination 1; B: Combination 2; C: Combination 3; D: Combination 4

**Figure 4:**
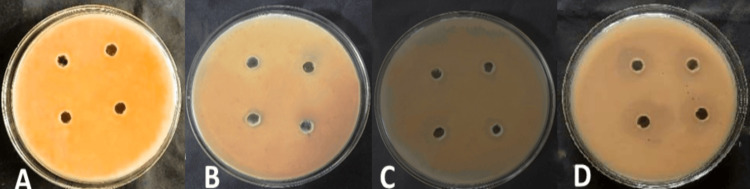
Antifungal activity of different combinations of neem leaf extract with Eucalyptus citriodora oil and Cymbopogon martini oil on Trichophyton tonsurans. A: Combination 1; B: Combination 2; C: Combination 3; D: Combination 4

Figures 3, 4 show the effects of different combinations of neem leaf extract with selected oils against *Microsporum canis *and *Trichophyton tonsurans*, respectively.

When the results from each well were compared (Tables 3, 4), it was seen that most growth inhibition was achieved when neem leaf extract was mixed with (0.5-0.8 μl) of *Eucalyptus citriodora* oil and (0.5-1.5 μl) of *Cymbopogon martini* oil. It was observed that, when low doses of each oil and neem leaf extract were combined together, a notable zone of inhibition was seen as compared to the zone of inhibition observed for neem leaf extract and selected oils when evaluated individually.

Tables 4, 5 demonstrated that neem leaf extract and selected oils exhibited growth inhibition at the minimal inhibitory concentrations of 187.5 µg/ml for neem leaf extract, 0.625 μl/ml for* Eucalyptus citriodora* oil, and 1.25 μl/ml for *Cymbopogon* *martini *oil on *Microsporum canis*, and 375 µg/ml for neem leaf extract, 2.5 μl/ml for *Eucalyptus citriodora* oil, and *Cymbopogon martini* oil on *Trichophyton tonsurans*, respectively. 

**Table 4:** MIC for neem leaf extract and selected oils against Microsporum canis No growth: ---; growth: +++; MIC=minimum inhibitory concentration; NLE=neem leaf extract; CMO=*Cymbopogon martini* oil; ECO=*Eucalyptus citriodora* oil; STD= Fluconazole as standard (0.25µg/ml); CTRL=control (broth); N/A =not applicable

S. No.	NLE (µg/ml)	Remark	ECO (µl/ml)	Remark	CMO (µl/ml)	Remark
1	3000	---	80	---	80	---
2	1500	---	40	---	40	---
3	750	---	20	---	20	---
4	375	---	10	---	10	---
5	187.5	---	05	---	05	---
6	93.75	+++	2.5	---	2.5	---
7	46.87	+++	1.25	---	1.25	---
8	23.43	+++	0.625	---	0.625	+++
9	11.71	+++	0.312	+++	0.312	+++
10	5.86	+++	0.156	+++	0.156	+++
11	2.92	+++	0.078	+++	0.078	+++
12	STD	---	N/A	N/A	CTRL	+++

**Table 5:** MIC for neem leaf extract with concentrations of oils against Trichophyton tonsurans No growth: ---; growth: +++; MIC=minimum inhibitory concentration; NLE=neem leaf extract; CMO=*Cymbopogon martini *oil; ECO=*Eucalyptus citriodora* oil; STD=Fluconazole as standard (0.50µg/ml); CTRL=control (broth); N/A=not applicable

S. No.	NLE (µg/ml)	Remark	ECO (µl/ml)	Remark	CMO (µl/ml)	Remark
1	3000	---	80	---	80	---
2	1500	---	40	---	40	---
3	750	---	20	---	20	---
4	375	---	10	---	10	---
5	187.5	+++	05	---	05	---
6	93.75	+++	2.5	---	2.5	---
7	46.87	+++	1.25	+++	1.25	+++
8	23.43	+++	0.625	+++	0.625	+++
9	11.71	+++	0.312	+++	0.312	+++
10	5.86	+++	0.156	+++	0.156	+++
11	2.92	+++	0.078	+++	0.078	+++
12	STD	---	N/A	N/A	CTRL	+++

## Discussion

The utilisation of herbal remedies for the ailments of numerous skin diseases dates back to ancient times. Even in the era of medical advancements, the utilisation of herbs in aromatherapy is widely accepted. Herbal medicines have a strong potential to replace synthetic antimicrobials, which proved to be less effective due to microbial resistance.

Several authors have confirmed "neem" and its compounds as a powerful and promising antifungal agent against medically important dermatophytes from the genera *Microsporum*, *Trichophyton,* and *Epidermophyton* that commonly cause skin disease [14,15]. Baby et al. [16] compiled a collection of recent studies and patents that address the effectiveness of neem in treating acne and other common skin disorders. The inhibitory effects of neem leaf organic extract and its constituents on several pathogenic fungi were demonstrated in earlier studies. As per the findings of Natarajan et al. [17] and Venugopal and Venugopal [18], neem seed extract effectively inhibited the growth of *Trichophyton mentagrophytes*, *Microsporum nanum*, and *Trichophyton rubrum* at a concentration of 31 µg/ml for seed oil extract, and 125-500 µg/ml for leaf extract; however, the authors also saw a decrease in the inhibitory zone of all dermatophytes under study during a 30-day period when the neem seed extract was incorporated in the media. A study using aqueous, ethanolic, and ethyl acetate extracts of neem leaves when tested against important human pathogens concluded that triterpenoids present in the neem leaf extract are exclusively responsible for the antifungal activity, which showed additive effects when used in combination rather than their individual effects [19]. A similar study by Musyimi et al. [20] confirmed the high potency of *Eucalyptus citriodora* against *Microsporum* *gypseum*, *Trichophyton tonsurans,* and *Candida albicans,* while Gemeda et al. [21] also proved the effectiveness of essential oils and their formulations of *Cymbopogon martini* oil against non-dermatophytes, including *Microsporum canis*. The prospective antifungal properties of a combination of *Azadirachta indica *(neem) oil, *Eucalyptus citriodora* oil, *Cymbopogon martini* oil, and neem leaf extract on medically significant dermatophytes were examined by Tiple et al. [22]. Scientific literature also shows that essential oils from *Eucalyptus citriodora*, *Cymbopogon martini*, and *Azadirachta indica* (neem) are beneficial in treating several fungal diseases.

In the previous studies, the antifungal activities were carried out either on neem leaf extracts or the selected herbal oils individually on *Microsporum canis* and some fungi from the *Trichophyton *genera, but not in combination with oils and extracts. However, no such study has been reported on the stated combination against *Trichophyton* *tonsurans*, a common causative fungus for tinea capitis.

Hence, with the objective of evaluating the antifungal effectiveness of selected oils in combination with neem leaf extract against dermatophytes responsible for serious skin infections such as tinea capitis, an in-vitro evaluation study was carried out. The study showed promising results in inhibiting the growth of *Microsporum canis* at MICs of 187.5 μg/ml for neem leaf extract and 0.625 and 1.25 μl/ml for *Eucalyptus citriodora* oil and *Cymbopogon martini* oil, respectively (Table 5). Moreover, when the much lower dose (50 µg/ml neem leaf extract) was mixed with the combination of two selected oils, 0.5µl of *Eucalyptus citriodora *oil and 0.5µl of *Cymbopogon martini* oil, a visible inhibition of growth was observed, and it became more prominent as the concentration was further increased (Table 3, Figure 3). It was seen as good as compared to the effect of standard Fluconazole. Similarly, the result evidenced that growth inhibition of *Trichophyton tonsurans* was observed at MICs of 375 μg/ml for neem leaf extract and 2.5 μl/ml for *Eucalyptus citriodora* oil and *Cymbopogon martini* oil, respectively (Table 6). However, when the much lower dose (100 µg/ml) of neem leaf extract was mixed with the combination of these selected oils (0.5µl of *Eucalyptus citriodora* oil and 1.0µl of *Cymbopogon martini* oil), a visible inhibition of growth was observed, and it became more prominent as the concentration was increased further (Table 4, Figure 4).

It is known that the antifungal effects of neem leaf extract and oils against dermatophytes are connected to their chemical makeup. Specifically, the terpenoids such as nimbin, nimolol, and nimbidin, which are extensively found in the neem leaves as well as the two major oxygenate monoterpenes citronellal and citronellol, which are abundantly found in *Eucalyptus citriodora *oil, and the most abundantly present trans-geraniol, which makes up 60.9% of *Cymbopogon martini* essential oil, are responsible for the antifungal effect. The study was limited to only two species of fungi; it can be done on other species of genera *Microsporum, Trichophyton, *and *Epidermophyton* responsible for tinea infections.

Although the minimum inhibitory concentrations (MICs) of selected oils and neem leaf extract are higher than those of the conventional drug fluconazole, it is important to consider the significant adverse effects of synthetic medicines such as azoles. The results of this study demonstrated a new investigation into the therapeutic potential of neem in combination with *Eucalyptus citriodora* and *Cymbopogon martini* oils for the management of the most commonly found microorganisms responsible for most of the cases of tinea capitis. *Trichophyton tonsurans*, which is prevalent in most of the cases of tinea capitis, was studied for the first time. 

## Conclusions

The selected oils confirmed prominent inhibitory effects against the causative dermatophytes when combined with neem leaf extract. It is concluded that *Microsporum canis *and *Trichophyton tonsurans*, responsible for the majority of cases of tinea capitis, are sensitive to neem leaf extract at moderate concentrations and show species-specific sensitivity to the oils under study. The findings of the current study state that, when neem leaf extract was studied together in a combination with the selected oils of *Eucalyptus citriodora *and *Cymbopogon martini* below their minimum inhibitory concentration (MIC) values, the entity exhibited a significant zone of fungal growth inhibition. The effect of combining neem leaf extract with selected oils was found to be stronger than their individual effects. It seems that a new chemical profile was formed when the oils are mixed, which has a different range of fungitoxicity than the individual oils. This suggests the possible occurrence of a synergistic phenomenon that might be due to multimodal action. It is crucial to incorporate the neem leaf extract with selected oils in a suitable topical formulation to treat the increasing incidence of opportunistic fungal skin infections, particularly after the COVID-19 pandemic era.
